# The MicroRNA Family Gets Wider: The IsomiRs Classification and Role

**DOI:** 10.3389/fcell.2021.668648

**Published:** 2021-06-09

**Authors:** Luisa Tomasello, Rosario Distefano, Giovanni Nigita, Carlo M. Croce

**Affiliations:** Department of Cancer Biology and Genetics, Comprehensive Cancer Center, The Ohio State University, Columbus, OH, United States

**Keywords:** microRNA, variants, isomiRs, microRNA biogenesis, novel microRNA in cancer

## Abstract

MicroRNAs (miRNAs or miRs) are the most characterized class of non-coding RNAs and are engaged in many cellular processes, including cell differentiation, development, and homeostasis. MicroRNA dysregulation was observed in several diseases, cancer included. Epitranscriptomics is a branch of epigenomics that embraces all RNA modifications occurring after DNA transcription and RNA synthesis and involving coding and non-coding RNAs. The development of new high-throughput technologies, especially deep RNA sequencing, has facilitated the discovery of miRNA isoforms (named isomiRs) resulting from RNA modifications mediated by enzymes, such as deaminases and exonucleases, and differing from the canonical ones in length, sequence, or both. In this review, we summarize the distinct classes of isomiRs, their regulation and biogenesis, and the active role of these newly discovered molecules in cancer and other diseases.

## Introduction

MicroRNAs are small non-coding RNA observed for the first time in the early 1990s (Lee et al., 1993) and characterized as a class of functional molecules in *Caenorhabditis elegans*, 10 years later (Reinhart et al., 2000; Lagos-Quintana, 2001). The discovery of microRNAs emphasizes the role of RNA as a functional molecule regulating gene expression at the post-transcriptional level (Huntzinger and Izaurralde, 2011). More than 2,000 (2,654, according to miRBase v22) mature microRNAs have been discovered in *Homo sapiens* (Kozomara et al., 2019). Several studies have elucidated the relevance of these molecules in regulating cellular processes and their steady presence in physiological and disease-related pathways (Friedman et al., 2008).

The microRNA maturation is a multi-step processing event that starts in the nucleus. The RNAse III DROSHA, in connection with the Microprocessor complex subunit DGCR8 (DiGeorge syndrome critical region 8), cleaves a primary RNA-transcript into a stem-loop precursor of approximatively 70 nucleotides, called pre-miRNA. This RNA-hairpin product is carried into the cytoplasm by Exportin 5 (XPO5) and processed by the RNAse III DICER into the mature microRNA (Ha and Kim, 2014).

Once the microRNA biogenesis is complete, the single-strand mature molecule is loaded by the RISC (RNA-induced silencing) complex. The mRNA target recognition occurs through the binding between the short seed region at the 5′ of the microRNA (nucleotides 2–8) and a partially or perfectly complementary region on the target gene 3′ UTR (Ha and Kim, 2014).

At least 45,000 sequences matching with microRNA seed sequences, the miRNA responsive elements (MRE), were found in 3′ UTR of human protein-coding genes (Friedman et al., 2008), indicating that these small RNAs could regulate most of the human proteins (Friedman et al., 2008). Due to the seed-sequence brevity, it is conceivable to predict more than one target for each microRNA. Indeed, hundreds of mRNAs could be controlled by a single microRNA (Friedman et al., 2008).

Epitranscriptomics is the study of post-synthetic modifications involving the RNA chemical structure (Frye et al., 2016). These changes, mediated by a wide range of proteins, including but not limited to RNA-methyltransferases, deaminases, uridyltransferases, poly(A) RNA polymerases, and exonucleases (De Almeida et al., 2018; Lan et al., 2019; Yu and Kim, 2020), also occur on microRNAs (Alarcón et al., 2015; Nishikura, 2016; Gutiérrez-Vázquez et al., 2017) and could be responsible for their sequence and length changes.

The rise of the high-throughput technology next-generation sequencing (NGS) has recently allowed several novel microRNAs to be detected alongside the well-known sequences. At first, these new molecules were interpreted as sequencing/mapping errors. However, later on, it was widely demonstrated that the percentage of *non-templated* nucleotide additions (%NTA) observed in small RNA sequencing data was significantly higher than the expected rate of sequencing error-rate calculated using small artificial RNAs (Linsen et al., 2009; Wyman et al., 2011). The development of more advanced analysis algorithms has supported these studies in confirming that canonical microRNA sequence modifications are not experimental artifacts but physiological events occurring *in vivo* (Linsen et al., 2009; Wyman et al., 2011). Moreover, there is evidence that isomiRs have a functional role just as their related canonical fragments: microRNA isomers can bind Argonaute (Ago) proteins, as demonstrated by co-immunoprecipitation assay (Cloonan et al., 2011; Londin et al., 2015; Haseeb et al., 2017), and can inhibit the expression of specific targets, as shown by luciferase assay *in vitro* (Cloonan et al., 2011).

In 2015, Londin et al. (2015) identified 3,707 novel microRNAs examining 1,323 samples from 13 different human tissues. The data presented on these newly discovered molecules suggested that, as the canonical microRNAs, the novel isoforms have a tissue-dependent expression (Londin et al., 2015). Their genome distribution is mostly intergenic (57.6%) and intronic (17.4%); moreover, out of the 31 miRNA genomic clusters identified by the authors, 21 involved novel variants, further proving a similar genomic organization with the canonical counterpart (Londin et al., 2015).

MicroRNA isoforms are heterogeneous and can variate for length, sequence, or both. The sequence variants hold more or fewer nucleotides at 5′ or a 3′ end than the canonical ones. Concurrently, the polymorphic (internal) isomiRs include different nucleotides within the mature sequence that distinguish these isoforms from the database-annotated microRNAs (Wu et al., 2018).

**FIGURE 1:**
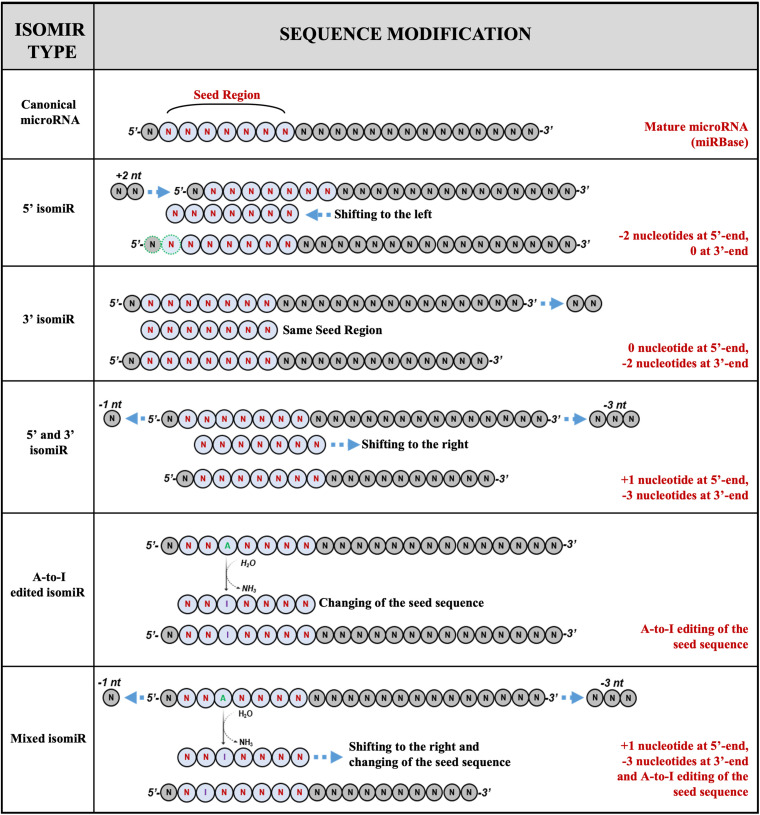
Examples of isomiRs. MicroRNA isoforms can variate for length, sequence, or both. The current classification identified five classes of variants: (1) canonical microRNAs; (2) 5′ isomiRs; (3) 3′ isomiRs; (4) polymorphic isomiRs; (5) mixed type isomiRs.

A recent classification categorizes the microRNAs and their variants into five classes (Figure 1):

(a) canonical microRNAs, whose mature sequence is the one reported in the microRNA databases;
(b) 5′ isomiRs, with changes in length at the 5′ end;
(c) 3′ isomiRs, with changes in length at the 3′ end;
(d) polymorphic isomiRs, with identical length except for changes within the mature sequence, between the first and the last nucleotide; and
(e) mixed type isomiRs, with changes in length and sequence (Wu et al., 2018).

Small variations in length and sequence of mature microRNA could be responsible for seed modification, potentially resulting in targetome shifting. This molecular event implies that isomiRs could have distinct or divergent functions compared with their related canonical counterparts. IsomiR expression varies among different tissue and cancer types, demonstrating their functional peculiarity and potential role as biomarkers (Telonis et al., 2017). Given the critical gene-regulatory function of these small molecules, their diffuse expression, and their involvement in the control of cellular processes, it is crucial to acquire a comprehensive knowledge of all the microRNAs and their functional isoforms expressed.

## 5′ and 3′ Isomirs

MicroRNAs with modifications of the sequence are called 5′ or 3′ isomiRs, depending on which microRNA end shifts. The 5′ isomiR rate is significantly lower than the 3′ isomiR one: 5–15% compared with 40–50% (Tan et al., 2014), even if the low percentage of 5′ variants can be offset by a high expression of these new isoforms and still have a relevant impact on the regulation of shared or exclusive targets (Chiang et al., 2010). It would be logical to assume that variations occurring at the 5′ end of the mature microRNA, which could reasonably affect the seed sequence, should weigh more on the potential targetome shifting than variations involving the 3′ end. Nevertheless, it was proven that the pairing between the microRNA 3′ end and its target firmly contributes to the interaction stability, maintaining favorable total interaction energy. The microRNA 3′ end plays a compensatory role when the presence of mismatches or bubbles between the mRNA target and the microRNA-seed region makes the binding weak (Bail et al., 2010; Moore et al., 2015).

Variations in length could be the consequence of DROSHA and DICER imprecise cleavage during the microRNA biogenesis steps or the action of specific exonucleases that remove nucleotides at its extremities, making the microRNA shorter (Neilsen et al., 2012). In both cases, the resulting isomiRs are classified as *templated* because their sequences match the parental gene (Neilsen et al., 2012). The length differences can also be attributed to the post-transcriptional addition of few nucleotides at the 5′ or 3′ end of the mature sequence by nucleotidyl transferases (Wyman et al., 2011). These variants are considered *non-templated* because they contain nucleotides not existing in the parental gene sequence (Neilsen et al., 2012).

### DROSHA and DICER Alternative Cleavage: From One Pri-miRNA Gene to Several MicroRNA Variants

**FIGURE 2:**
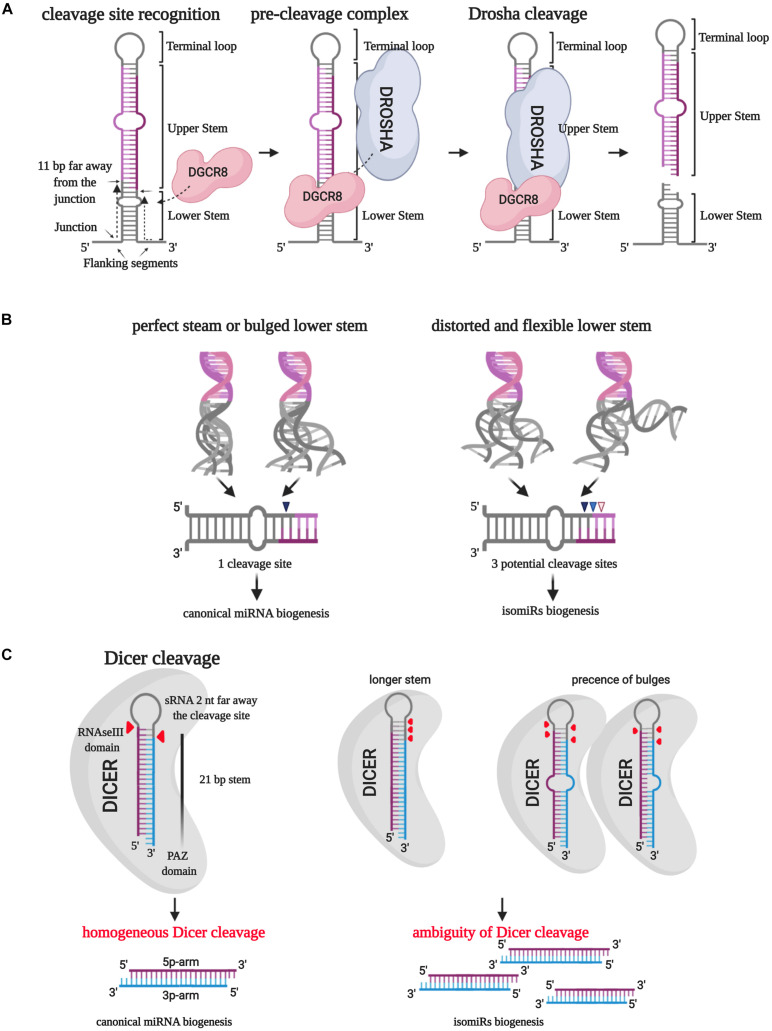
IsomiR biogenesis. **(A)** The RNAse III DROSHA, aided by DGCR8, processes pri-miRNA in the nucleus and produces the first cut in correspondence of the 5′ end of the 5p arm and 3′ end of the 3p arm. DGCR8 acts as a molecular meter and identifies the cleavage site 11 bp far away from the junction point between the lower stem and the basal unpaired sequences. **(B)** The secondary structure of the lower stem of pri-miRNA affects the DROSHA cleavage precision: a perfect or bulged lower stem leads to a homogeneous cleavage site in more than 97% of the cases. On the contrary, a distorted and flexible lower stem creates three potential cleavage sites. **(C)** The RNase III DICER processes the short hairpin RNA (shRNA) by eliminating the terminal loop and forming a double-strand miRNA/miRNA*. Different lengths and the presence of bulges can affect the PAZ domain-mediated “measurement” of the lower stem leading to the selection of multiple cleavage sites.

The biogenesis of microRNAs starts with transcribing a primary structure (pri-miRNA) by RNA polymerase II. The pri-miRNA consists of a terminal loop, an upper and a lower stem surrounded by two basal single-strand flanking sequences (Ha and Kim, 2014). The RNAse III DROSHA (Table 1), aided by DGCR8, processes this molecule in the nucleus and produces the first cut in correspondence of the 5′ end of the 5p arm and 3′ end of the 3p arm (Ha and Kim, 2014). Han et al. (2006) described how the DROSHA cleavage is always expected to occur 11 bp far away from the junction between the stem and the basal unpaired sequences (ssRNA/dsRNA junction). The precision of this phenomenon induced to hypothesize that DGCR8 could act as a “molecular meter,” recognizing and anchoring the pri-miRNA substrate, forming the “pre-cleavage complex,” and preparing the way for DROSHA-mediated catalysis (Han et al., 2006; Figure 2A). Making mutant artificial pri-miRNA-30a with modified regions, they demonstrated that the terminal loop does not affect the cut because it weakly interacts with DGCR8 protein. However, modifications in this pri-miRNA area, especially in the loop size, could compromise the catalysis efficiency (Han et al., 2006). Besides, alterations of the stem length and the single-stranded basal segments could undermine the cleavage site recognition from DGCR8, leading to imprecise processing of the pri-miRNA (Han et al., 2006).

**TABLE 1:** Enzymes affecting microRNA length and sequence.

Name	Type	References
DROSHA	Ribonuclease (RNase) III double-stranded RNA-specific	Han et al., 2006; Starega-Roslan et al., 2015; Bofill-De Ros et al., 2019
DICER1	Ribonuclease (RNase) III double-stranded RNA-specific	MacRae et al., 2007; Gu et al., 2012; Ha and Kim, 2014; Starega-Roslan et al., 2015; Song and Rossi, 2017
Nibbler	3′–5′ exonuclease	Han et al., 2011; Liu et al., 2011; Wyman et al., 2011
PARN	3′-exonucleases with a preference for poly(A) substrates	Katoh et al., 2015
TENT2 or PAPD4 or GLD2	Poly(A) RNA polymerase	Katoh et al., 2009; Burroughs et al., 2010; Wyman et al., 2011
TUT4 or ZCCHC11	RNA uridyltransferase	Jones et al., 2009; Wyman et al., 2011; Thornton et al., 2014; Yang et al., 2020
TUT3 or PAPD5	Poly(A) RNA polymerase	Wyman et al., 2011
MTPAP or TENT6	Mitochondrial poly(A) polymerase	Wyman et al., 2011
PAPOLG	Poly(A) DNA/RNA polymerase	Katoh et al., 2009
TUT1 or TENT1	Terminal uridylyltransferase and nuclear poly(A) polymerase	Wyman et al., 2011
TUT7 or ZCCHC6 or PAD6	Terminal uridylyltransferase	Wyman et al., 2011; Thornton et al., 2014; Yang et al., 2020
ADAR enzymes	Adenosine deaminase RNA specific	Bazak et al., 2014; GTEx Consortium et al., 2017; Wang et al., 2017; Cesarini et al., 2018; Li et al., 2018; Xu et al., 2019; Marceca et al., 2020
APOBEC enzymes	Cytidine deaminase RNA specific	Marceca et al., 2020

Bofill-De Ros et al. (2019) explained how the 3D structural characteristics of pri-miRNA affect the DROSHA cleavage ambiguity (Figure 2B). The employment of miR-9 paralogs showed that the pri-miRNA lower-stem flexibility and distortion could play a central role in driving DROSHA cleavage, potentially destabilizing the fidelity of the cut (Bofill-De Ros et al., 2019).

After the pre-miRNA exportation from the nucleus to the cytoplasm by Exportin-5 (XPO5), another RNAse III, named DICER (Table 1), processes the short hairpin RNA (shRNA) by eliminating the terminal loop and forming a double-strand miRNA/miRNA^∗^ (Ha and Kim, 2014). Similarly to the first catalysis, the cleavage precision is essential to generate a specific mature molecule. Indeed, the cut inaccuracy could generate new microRNA variants with altered seed sequences and, reasonably, different targets and roles (Tan et al., 2014).

DICER is an RNAse III enzyme holding eight different domains, including an amino-terminal helicase domain, a PAZ (Piwi/Argonaute/Zwille) domain, and 2 RNase III domains (Song and Rossi, 2017). DICER-mediated pre-miRNA catalysis starts with recognizing the open ends of pre-miRNA and trapping the RNA molecules inside the enzyme catalytic pocket. At this step, the PAZ domain is essential to “measure” the dsRNA from the 3′ end of the shRNA to ensure the generation of a mature microRNA duplex with a species-specific length and the typical 2-nucleotides 3′ overhang (MacRae et al., 2007).

Gu et al. (2012) employed artificial shRNAs to describe the DICER processing of pre-miRNA. They established a “loop-counting rule” to predict the accuracy of the cut: DICER cleavage fidelity can be maintained if the enzyme recognizes an ssRNA sequence, such as the terminal loop or an internal bulge, precisely situated two nucleotides far away from the cleavage site, previously determined by the PAZ domain “measuring.” In other words, the presence of a single-stranded structure in correspondence with the enzyme helicase domain is required to stabilize the catalytic RNAse III domain, thus supporting the correct cleavage (Gu et al., 2012; Figure 2C).

The fidelity of DROSHA and DICER cleavage is influenced not only by the pri-miRNA and pre-miRNA structures but also by their sequences. Deep sequencing data on the human cell line HEK293T, embryonic stem cells, and differentiated cells from murine models showed that DROSHA and DICER cleavage sites seldom include G residues on their sequences. Moreover, data highlighted a strong presence of U residues at both the mature microRNA ends (Starega-Roslan et al., 2015).

Although the study shows that the DROSHA cut fidelity seems more influenced by the cleavage site sequence than DICER, it was demonstrated that both the enzymes undergo an adequate sequence-dependent regulation that affects the precision of the cut, involving their RNAse domains differently. The DROSHA RIIIA domain produces more heterogeneous molecules than the DICER RIIIA domain, which cuts more precisely. On the contrary, the DROSHA RIIIB domain catalysis activity is much more specific than the DICER RIIIB domain one (Starega-Roslan et al., 2015).

Summing up, even as we are still used to considering isomiRs generation as the exception to the rule, this is a misconception: it is very infrequent that DROSHA and DICER cleavage produces only one microRNA variant from a single microRNA gene.

### IsomiRs Can Arise From Exoribonuclease Nibbling Activity

The trimming action of exoribonucleases (Table 1) could also be a source of microRNA variants (Han et al., 2011; Katoh et al., 2015). These enzymes act on mature microRNA ends or microRNA precursors during microRNA biogenesis processes. One of the first indications of this mechanism was observed in *Drosophila melanogaster*, where the 3′–5′ exoribonuclease Nibbler (Nbr) (Table 1) contributes to generating a 22-nt-long microRNA after the processing mediated by DICER (Han et al., 2011). Han et al. (2011) studied the case of miR-34 in flies: the maturation of this microRNA runs through the typical multi-step DROSHA/DICER biogenesis process. DICER can generate molecules of 22 or 24 nt, and Ago1 or Ago2 can load both of them. In the former case, the RISC complex constitution led to the post-transcriptional regulation of microRNA targets. In the latter case, the longer molecules bond to Ago1 is available for the Nibbler trimming because of the weaker binding with the Ago1 PAZ domain. The sculpt of the 3′ end and the restoration of a 22-nt-long molecule enhances the activity of miR-34 (Han et al., 2011). *Nibbler* knockout causes the loss of many 3′ isomiRs and a semi-lethal and sterile phenotype in flies (Han et al., 2011; Liu et al., 2011).

Experiments performed on the human cervical carcinoma cell line HeLa have shown a microRNA 3′ variability tracing the one observed in *D. melanogaster*, thus suggesting the presence of a human exoribonuclease homolog of Nibbler (Han et al., 2011).

Katoh et al. (2015) investigated the role of another exoribonuclease named PARN, which interacts with microRNAs, specifically miR-122, in hepatocellular carcinoma cells. CUGBP1, a protein binding UG-rich microRNAs, recruits PARN and leads it to the miR-122. PARN causes deadenylation, with consequent destabilization of the miR-122 3′ end, affecting the cellular level of canonical miR-122 (Katoh et al., 2015).

To date, the definition of the role of exoribonucleases in the human isomiR generation is still at the beginning. Nevertheless, the evidence collected so far suggests the likely presence of mammalian homologs with an active role in isomiRs biogenesis and regulation of mature microRNA stability.

### Non-templated microRNA Variant Generation by Nucleotidyl Transferases

The post-transcriptional addition of nucleotides to small RNA 3′ end contributes to the heterogeneity of microRNAs and the generation of new variants. Through next-generation small RNA sequencing experiments, Wyman et al. (2011) defined 39 microRNA modifications ascribable to 3′ nucleotides addition. These modifications are physiological and influenced by biological processes, such as cell differentiation (Berezikov et al., 2006; Wyman et al., 2011). They were recognized in a broad range of species and cell types, in different diseases and biological conditions (Berezikov et al., 2006). The most prevalent modifications identified are adenylation and uridylation. In human and mouse, ∼50% of 3′ modifications are mono-adenylation, and ∼25% are mono-uridylation (Wyman et al., 2011). The nucleotide additions, mediated by at least eight nucleotidyl transferases, affect microRNA stability and efficiency undergoing their modification process (Jones et al., 2009; Katoh et al., 2009; Burroughs et al., 2010; Wyman et al., 2011). Typically, microRNA uridylation is associated with molecule degradation, whereas adenylation leads to improved microRNA stability (Rüegger and Großhans, 2012). The principal nucleotidyl transferases identified so far are PAPD4 (TENT2 or GLD2), ZCCHC11 (TUT4), PAPD5 (TUT3), MTPAP (TENT6), PAPOLG, TUT1 (TENT1), and ZCCHC6 (TUT7) (Jones et al., 2009; Katoh et al., 2009; Burroughs et al., 2010; Wyman et al., 2011; Table 1). The downregulation of these enzymes contributes to a specific decreased number of microRNA 3′ end modifications. For example, the depletion of TUT1 and ZCCHC6 causes the selective loss of the 3′ U variant of the miR-200a and let-7e, respectively (Wyman et al., 2011). Zcchc11 (TUT4) and Zcchc6 (TUT7) modify, through 3′ uridylation, a specific microRNA group that shares a TUTase recognition sequence motif and targets proteins belonging to the Homeobox family in P19 embryonal carcinoma cells from mouse (Thornton et al., 2014). Thornton et al. (2014) have further demonstrated the importance of these proteins during zebrafish development steps through the regulation of Homeobox proteins, emphasizing that microRNA uridylation is a physiological and finely regulated process. Katoh et al. (2009) described a delicate mechanism, orchestrated by the nucleotidyl transferase GLD2 and exonuclease enzymes. The process stabilizes the microRNA-122 molecule, with specific liver-associated functions in hepatocellular carcinoma cells. After the canonical biogenesis process, the 22-nt variant of miR-122 is stretched at the 3′ end by GLD-2-mediated poly(A) adenylation. Later, this longer variant undergoes cleaving by 5′–3′ exonucleases that restore a molecule long between 21 and 23 nucleotides. This elongation/degradation process “corrects” the microRNA length to produce a stable molecule, not too long, not too short, that can be loaded by Ago2 (Katoh et al., 2009). Similarly, other microRNAs, including but not limited to miR-7, miR-222, and miR-769, are subjected to uridylation by TUT4 and TUT7 when the binding with Ago2 leaves their 3′ end exposed (Yang et al., 2020). Then, oligouridylated microRNAs undergo degradation by exonuclease DIS3L2 (Yang et al., 2020).

These represent a few examples that can describe how the addition of *non-templated* nucleotides regulates the stability or degradation of microRNAs, generating new isomiRs and indirectly changing their mRNA targets expression pattern.

## Polymorphic Isomirs

### SNPs in microRNAs

The frequency of single-nucleotide polymorphisms (SNPs) occurring within microRNA genes is consistently lower than observed in other genomic regions (Saunders et al., 2007). The selective evolutionary pressure on microRNA sequences deters genetic variations on microRNA loci, supporting the conservation of these regions and their functional importance. The SNPs’ density observed in microRNA seed sequences is less than 1% of the total SNPs in the human genome (Saunders et al., 2007). Despite the rareness of this event, genetic variations in precursor or mature molecule sequences significantly impact microRNAs transcription and biogenesis. The presence of SNPs could block or enhance some microRNA maturation, thus changing canonical and new microRNA variants expression patterns and influencing their gene-silencing regulation (Sun et al., 2009). To cite a few examples, Calin et al. (2005) demonstrated that a germline-specific mutation on the primiR-15a/16-1 impairs the tumor suppressor miR-16-1 biogenesis, thus increasing the risk of familial chronic lymphocytic leukemia.

Similarly, the SNP rs895819 on the terminal loop of pre-miR-27a blocks the derived microRNA maturation, whose down-regulation is associated with an attenuating risk of familial breast cancer (Yang et al., 2010). In 2012, a genome-wide study (Gong et al., 2012) compared the canonical pri-miR, pre-miR, and mature microRNA sequences annotated on the miRBase database (Griffiths-Jones, 2006) from 9 species with the classified SNPs collected from the NCBI dbSNP database (Sherry, 2001). Predictions of putative targets for wild-type microRNAs revealed that more than 50% of predicted miRNA–target bindings (55,887) were negatively affected by SNPs in the seed sequence. At the same time, more than 50% of the predicted targets for the new microRNA variants were exclusive for the SNP-isomiRs (Gong et al., 2012). Experimental validation of these data through Luciferase assay showed a partial or total loss of binding for at least four isomiRs and the addition of a new target for miR-627 (Gong et al., 2012). Particularly impressive is the SNP rs3746444 on miR-499-3p that heavily affects the binding between this isomiR and BCL2, which remains a validated target for the canonical molecule (Gong et al., 2012). Another example is the case of miR-124, whose isomiR, holding the SNP rs34059726, completely loses the ability to target ATP6V0E1, restoring the Luciferase activity from 10% in the presence of wild-type microRNA to 80% (Nishikura, 2016).

The importance of SNPs is defined not only by their frequency but also by the functional modifications they could induce. In the case of microRNAs, it has been well established that the presence of SNPs on microRNA genes sequence could affect their biogenesis and, indirectly, the expression of their targets. We have recently started to associate the presence of SNPs within mature microRNAs with the generation of isomiRs. Small RNA sequencing allowed the discovery of several new microRNA variants ascribable to genetic variations. The characterization of these SNP-isomiRs and the potential role that they could assume in specific biological conditions and diseases remains an exciting field to explore in the next future.

### MicroRNA Editing

RNA editing is a type of RNA processing that occurs on double-strand RNA molecules at the co-transcriptional or post-transcriptional level (Nishikura, 2016). This process consists of specific bases deamination leading to a modification of the sequence (Nishikura, 2016). Among observed editing types, the adenosine-to-inosine (A-to-I) and the cytosine-to-uracil (C-to-U) (Nishikura, 2016) represent the most common modifications. The A-to-I modification contributes to almost 90% of all editing events, and it is mediated by proteins belonging to the Adenosine Deaminase Acting on RNA (ADAR) family (Table 1), particularly ADAR (or ADAR1) and ADARB1 (or ADAR2) (Bazak et al., 2014). The interpretation of the inosine as guanine *de facto* results in a functional substitution A-to-G (Bazak et al., 2014).

To date, 2,885 A-to-I and 104 C-to-U unique editing events have been identified on microRNA transcripts, but only 257 have been confirmed by further investigations (Marceca et al., 2020). The consequences of editing modifications on microRNAs could change their expression or function (Li et al., 2018). Editing events involving pri-miRNA, pre-miRNA, and mature sequences could affect the microRNA maturation process, interfering with DROSHA and DICER cleavage, as well as the asymmetric selection of the strand (Li et al., 2018).

The editing contribution to the isomiRs generation is substantial, even if the accurate detection of editing sites in mature microRNAs by small RNA sequencing could be a long and complicated process due to the brevity of these molecules (Li et al., 2018). Li et al. (2018) identified 367 new editing sites in mature microRNAs. Their data on edited pre-miRNAs allowed the development of a custom pre-miRNA database to map newly edited mature microRNAs correctly. The editing sites have been identified throughout the mature molecule sequence, anticipating the potential change of seed sequence in these microRNAs (Li et al., 2018). Indeed, their target prediction analyses demonstrated that canonical and edited microRNAs shared only 10–35% of common targets (Li et al., 2018).

A critical functional effect has been observed following the editing of miR-200b in position 5 (within the seed sequence) in breast and ovarian cancer cells (Wang et al., 2017). In these two cancer models, the editing of miR-200b induces the loss of ability to target ZEB1/ZEB2 (Wang et al., 2017). Concurrently, the gain of new targets, including the metastasis suppressor LIFR, contributes to conferring a new role to the edited miR-200b: a negative regulator of cancer metastasis becomes a promoter of cell invasion and migration in response to ADAR-mediated modifications (Wang et al., 2017).

Similarly, canonical and edited miR-589-3p play two distinct roles in normal brain and glioblastoma tissues (Cesarini et al., 2018). Cesarini et al. (2018) showed that almost 100% of miR-589-3p molecules are edited in normal brain cells. This editing level strongly decreases in tumor cells together with astrocytomas grade of malignancy. Moreover, they demonstrated that the edited miR-589-3p gains the capacity to inhibit ADAM12, a well-characterized oncogene promoting glioblastoma cell aggressiveness, thus explaining its high editing level in normal brain cells (Cesarini et al., 2018).

By contrast, Xu et al. (2019) demonstrated the tumor-suppressive effect of edited miR-379-5p in ovarian, breast, renal, and lung cancer cell lines. Relying on The Cancer Genome Atlas (TCGA) miRNA-Seq data, they observed a lower editing level of miR-379-5p in seven different tumor tissues, significantly correlating the higher expression of the edited variant with better patient survival. Experiments conducted *in vitro* and *in vivo* demonstrated that by acquiring a new group of targets, particularly CD97, edited miR-379-5p induces apoptosis in cancer cells and, consequently, reduces cell proliferation (Xu et al., 2019).

These findings suggest that microRNA editing is a critical event that could potentially affect the expression or the role of the edited molecules, whether it takes place within the seed sequence or other regions of precursors or mature molecules. Lastly, the editing level could become a predictive factor of risk when it causes loss or gain of microRNA function due to the targetome-shifting in some particular diseases.

## The Functional Importance of Isomirs

### IsomiRs Bind Ago Proteins

The Argonaute (Ago) proteins are essential mediators of microRNAs regulative action through suppressing protein translation or degrading mRNA by their specific RNase activity (Hutvagner and Zamore, 2002; Liu, 2004; Meister et al., 2004). Eight Ago proteins were discovered in humans, but only four can bind and load microRNAs, and just one, Ago2, holds the endonuclease activity essential to mediate the repressive action of microRNAs on their target (Meister et al., 2004). After the first step of biogenesis, the pre-miRNA hairpin is processed by the RNase DICER and then, as a mature microRNA molecule, loaded by the RISC Loading Complex (RLC), which comprises, in addition to DICER, TRBP and Ago2 (Gregory et al., 2004; Chendrimada et al., 2005).

Ago2 is identified as the RISC complex effector protein since it prevents the expression of the target mRNA via direct degradation or translation process barring (Liu, 2004; Meister et al., 2004). Therefore, it is considered the “slicer” of the RISC complex and the only protein essential for the complex proper functioning (Rand et al., 2004). It can be said that microRNAs exert their function of gene-expression repressors through their association with the Ago2 protein. Consequently, the evidence of microRNA recruitment by Ago2 strongly suggests the truly functional status of the microRNA molecule.

As previously mentioned, Londin et al. (2015) identified 3,707 novel microRNAs analyzing 1,323 samples across 13 different tissues. Crossing these data with 43 Ago CLIP-Seq (10 self-performed on their samples and 33 obtained from available public samples), they found 1,657 (44.7%) newly discovered miRNA sequences and 1,517 (54.7%) miRBase-cataloged microRNAs present in one or more of the samples examined, thus supporting the evidence of a similar microRNA-Ago binding rate for novel and canonical microRNAs.

In 2017, through AGO2 RIP-Seq analysis on normal and osteoarthritis chondrocytes, Haseeb et al. (2017) identified a pool of microRNAs and isomiRs, expressed in human chondrocytes, directly interacting with Ago2. MicroRNA novel variants represented 52% of all sequenced microRNAs (Haseeb et al., 2017). Although the authors detected isomiRs belonging to each of the categories (5′ or 3′ deletion, 5′ or 3′ addition, and internal substitutions), the approximate total of variants (46% out of 52%) was represented by 3′ isomiRs and only 6% by microRNAs with 5′ modifications (Haseeb et al., 2017). A reasonable explanation for reading this phenomenon is that Ago2 and the other RISC complex proteins bind the mature microRNAs in correspondence of the 5′ end, thus protecting microRNAs from exonucleases nibbling action (Haseeb et al., 2017).

In confirmation of an independent functional role for these Ago-bound isomiR, *in silico* target prediction analyses for the canonical miR-140-3p and one of the most abundant 5′ deletion isoforms have unveiled that they share only 50 targets out of 190 exclusive canonical and 317 exclusive isoform targets, suggesting a potential peculiar role for this 5′ isomiR in chondrocytes (Haseeb et al., 2017).

Not only comprehensive approaches proved the isomiR loading into Ago2 (Ebhardt et al., 2009; Martí et al., 2010). As in the case of 5′-isomiR-101 (Llorens et al., 2013) or the miR-222 isoforms (Yu et al., 2017), the study of specific novel variants has revealed, through Ago2 co-immunoprecipitation assays, the interaction between isomiRs and Ago2 protein.

Ultimately, the evidence of the cooperative binding between isomiRs and Ago2 has been repeatedly verified to support the hypothesis that isomiRs are functional molecules. They are likely to harness the same functional pathways and are loaded by the RISC complex as their canonical counterparts.

### The Target Redirecting

The most intriguing question about isomiRs is represented by their ability to repress new and different targets. In other words, are they unique and independent functional molecules? Cloonan et al. (2011) analyzed canonical microRNAs and their isomers expression in 10 different human tissues. The results demonstrated a strong correlation of expression between the two groups, corroborating the hypothesis that isomiRs could have a supportive role in targeting biological pathways already regulated by canonical counterparts (Cloonan et al., 2011). Moreover, their data suggested that canonical microRNAs and isomiRs cooperative targeting action is mainly geared toward key genes belonging to cancer pathways. The participation of more molecules in targeting only one mRNA strongly decreases the off-target effects (Cloonan et al., 2011).

In subsequent years, other groups have confirmed or contradicted these assumptions. Salem et al. (2016) studied the expression and function of miR-140-3p and its 5′ isomiR in breast cancer cells. Both these versions of miR-140-3p have been found upregulated in breast cancer tissues and participate in a tumor-suppressive strategy (Salem et al., 2016). Despite the collaborative repressing role, they affect different pathways: the canonical miR-140-3p controls the stemness of breast cancer cells, although the 5′ isoform, more expressed than the canonical microRNA, causes cell cycle arrest, along with inhibition of proliferation and cell migration. The seed sequence shifting in the 5′ variant allows the gaining of novel targets: COL4A1, ITGA6, and MARCKSL1 (Salem et al., 2016). In other cases, isomiRs and the canonical counterparts could have divergent functions, such as miR-411 and its 5′ isomiR in human vascular fibroblasts and venous tissues (van der Kwast et al., 2020): in response to acute ischemia, the level of 5′ isomiR-411 rapidly decreases, while canonical miR-411 undergoes upregulation (van der Kwast et al., 2020). Moreover, the seed sequence shifting contributes to the acquisition of exclusive targets for both microRNAs that could justify their opposite expression trend: only the canonical mir-411 represses the expression of TGFB, leading to a pro-angiogenic phenotype, while the 5′ isomiR controls F3 and ANGPT1, thus decreasing cell migration and angiogenesis (van der Kwast et al., 2020). Similarly, after neural differentiation in human embryonic stem cells, the 5′ isomiR-9-1 gains the capacity to repress two new targets, DNMT3B and NCAM2, concurrently losing the ability to inhibit CDH1, that persists as a canonical miR-9-1 target (Tan et al., 2014).

The study of miRNA expression in human retina samples led to identifying a new 5′ isoform of the neuronal-specific miR-124a-3p, representing less than 25% of mir-124a-3p total variants in human retina samples (Karali et al., 2016). Despite the shifting of one single base, the change of the seed sequence in this 5′ isoform supports the gain of a new target, CDH11, a gene involved in neuronal differentiation, never identified as a canonical miR-124a-3p target (Karali et al., 2016).

Not only 5′ isomiRs but also 3′ isomiRs can increase or diversify canonical microRNA functions, as described by Yu et al. (2017) for miR-222 and its longer 3′ variants. They have demonstrated how the upregulation of 3′ isomiR variations of miR-222 subverts the well-known anti-apoptotic role of canonical miR-222 by inhibiting many members belonging to the PI3K–AKT pathway such as PIK3R3 (Yu et al., 2017).

Together with the already cited examples of edited microRNAs, these findings illustrate that isomiRs are functional molecules that could extend or change the canonical microRNAs’ role by acquiring or losing different targets. On one side, the potential impact of 5′ isomiRs is readily explained by variations in the seed sequence and the resulting acquisition or loss of some target control. On the other side, the role of 3′ isomiRs is less predictable and more complex. Variations on the 3′ end of microRNAs could affect the molecule biogenesis or the degradation, the efficiency of the loading process by Ago proteins, and the stability and the strength of the miRNA:mRNA binding (Bofill-De Ros et al., 2020), thus creating or preventing the conditions for targets inhibition. The complicated rules governing the miRNA:mRNA binding could make the study of the isomiRs’ targeting properties long and frustrating. Most prediction bioinformatics tools rely on the conventional miRNA–target pairing recognition, considering the miRNA seed sequence solely. Therefore, the improvement of new strategies to discriminate selective targets for these novel microRNA variants is primary. In this context, the miR-CLIP technique (Imig et al., 2015), based on the transfection of pre-miRNAs conjugated with biotin and psoralen to trap mRNA targets in cells, aided the identification of targets differentially regulated by miR-124 and its 5′ isoform (Wang et al., 2020). After the transfection of a modified pre-miRNA-124 miR-CLIP probe in HEK293T cells, supported by the subsequent individual transfection of canonical and isomiR-124, the authors identified 16 potential targets (Wang et al., 2020). Out of these 16 selected candidates, 12 were mostly regulated by canonical miR-124, three were regulated by both isoforms, and just one was strongly inhibited exclusively by isomiR-124 (Wang et al., 2020).

In conclusion, it is plausible to assume that isomiRs have an independent targeting activity. They could play as supporters or competitors of canonical microRNAs, making the unbalanced biogenesis promoting an isoform expression instead of another (with identical or opposite functions) a protective cell strategy to strengthen or reduce a specific microRNA inhibition power.

## Techniques for Isomirs Detection

Next-generation (NextGen) sequencing is so far the method of choice for isomiR detection. The nature of the sequence to detect does not affect the efficiency or specificity of this technique because it is not based on the principle of primer or probe annealing, and indeed it is employed to discover new microRNA variants. The main obstacle preventing NextGen sequencing as a daily laboratory routine procedure is the cost and the need for most laboratories to rely on an external service, increasing experimental times.

**FIGURE 3:**
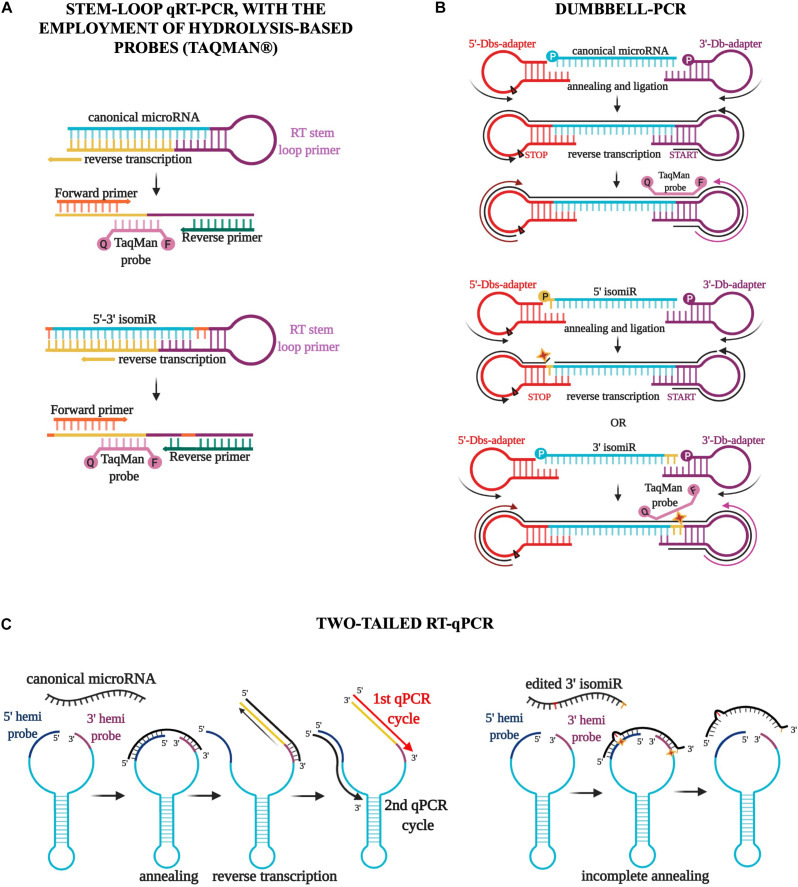
Three different qPCR techniques for the detection of isomiRs. **(A)** The stem-loop qRT-PCR, with the employment of hydrolysis-based probes (Taqman), has become the most commonly used commercial technique. However, this method has significant limitations in detecting and quantifying isomiRs accurately. The discrimination of two sequences that differ by only one or a few nucleotides is not guaranteed by this protocol and must be established empirically for each molecule using customized probes and appropriate controls. **(B)** Dumbbell-PCR employs a 3′-stem-loop adapter, which acts as the reverse transcription trigger, and a 5′-stem-loop adapter, which contains a stop signal for reverse transcription. IsomiR gaps or overlaps strongly impact the efficacy of the ligation process and the annealing of a Taqman probe partially complementary to the microRNA and partially to the 3′ adapter sequences. **(C)** Two-tailed RT-qPCR is characterized by the design of a long-structured primer (∼50 nucleotides) holding two hemiprobes complementary to the 5′ and 3′ ends of the microRNA. The reverse transcription starts from the 3′ end, extending the primer sequence with the complementary sequence of the target microRNA and simultaneously detaching the 5′ end. The amplification step uses two specific primers, one annealing the microRNA sequence and the other the 5′ hemiprobe. The use of two short hemiprobes increases the sensitivity and specificity of this technique: the brevity of these two sequences makes them more susceptible to possible isomiR mismatches.

Since microRNA research has assumed an even more critical role in molecular biology, many protocols for detecting these small RNAs have been developed (Ye et al., 2019). In particular, poly(A) and stem-loop qRT-PCR, with or without the employment of hydrolysis-based probes (Taqman) (Figure 3A), have become the most commonly used commercial techniques because of the high level of specificity, the brevity of experimental times, and the relatively low cost of reagents and machines (Ye et al., 2019). However, these methods have significant limitations in detecting and quantify isomiRs accurately. The annealing of primers and probes requires acknowledging the sequence, thus making the detection of new molecules technically impossible (Schamberger and Orbán, 2014; Magee et al., 2017). Moreover, the discrimination of two sequences that differ by only one or a few nucleotides is not guaranteed by these protocols and must be established empirically for each molecule using customized probes and appropriate controls (Schamberger and Orbán, 2014; Magee et al., 2017). It follows that the quantification of a specific isoform in a sample containing an abundance of the same microRNA variants is far from easy and is strongly affected by the expression of the particular isoform to detect (Avendaño-Vázquez and Flores-Jasso, 2020). Various attempts have been made recently to assess the expression of microRNA variants, including, for example, Dumbbell-PCR (Zhou et al., 2010; Honda and Kirino, 2015) and two-tailed RT-qPCR (Androvic et al., 2017). The first one is based on the employment of a 3′-stem-loop adapter, which acts as the reverse transcription trigger, and a 5′-stem-loop adapter, which contains a stop signal for reverse transcription (Honda and Kirino, 2015). The adapters are ligated specifically to the microRNA two ends by a T4 RNA ligase (Rnl2). Gaps or overlaps due to modifications of the microRNA sequence strongly influence the efficacy of this ligation process (Honda and Kirino, 2015; Figure 3B). Moreover, the employment of a Taqman probe partially complementary to the microRNA and partially to the 3′ adapter sequences confers further specificity to this method that should discriminate both 5′ and 3′ variations during the ligation and amplification steps (Honda and Kirino, 2015; Figure 3B). Two-tailed RT-qPCR is a technique based on the design of a long structured primer (∼50 nucleotides) holding two hemiprobes complementary, respectively to the 5′ and 3′ ends of the microRNA to detect (Androvic et al., 2017; Figure 3C). After the annealing between the long primer and the microRNA sequence, the reverse transcription starts from the 3′ end, extending the primer sequence with the complementary sequence of the target microRNA and simultaneously detaching the 5′ end (Androvic et al., 2017; Figure 3C). The following amplification step uses two specific primers, one annealing the microRNA sequence and the other the 5′ hemiprobe. The use of two short hemiprobes increases the sensitivity and specificity of this technique: the brevity of these two sequences makes them more susceptible to possible mismatches in the input sequence, as with isomiRs (Androvic et al., 2017; Figure 3C).

A different and more conventional approach has been considered to detect isomiRs containing edited nucleotides or SNPs. A mere Sanger sequencing of the RT-PCR products can determine the presence of modifications on pri-miRNA, pre-miRNA, or mature microRNA sequences (when required, after the addition of a poly(A) tail and a 5′ adapter to make the molecules long enough for the amplification step and following sequencing) (Kawahara, 2012). The limit of this technique is the ability to identify edited variants when representing less than 10% of the total. Moreover, it requires previous knowledge of the editing sites or SNPs, thus narrowing the discovery of new isoforms (Kawahara, 2012).

Multiplex Single Base Primer Extension Assay can also detect internal modifications on microRNA sequences (Podini and Vallone, 2009). This protocol starts with multiplex PCR amplification of the regions containing the modifications to identify. Purified PCR products are then amplified again using 5′→3′ primers with the last nucleotide at 3′ end adjacent to the modification and a fluorescently labeled dideoxynucleotide (ddNTP) corresponding to the modification site (Podini and Vallone, 2009). PCR products are loaded onto capillary electrophoresis, and the resulting electropherograms are analyzed with suitable analysis software (Podini and Vallone, 2009).

In conclusion, the heterogeneity of these molecules makes developing a reliable, easy-to-use, and universal protocol a challenge for molecular biologists. So far, a unique and extensively validated technique to detect isomiRs does not exist. However, although all these methods still suffer from specificity and efficiency limitations, even using nanomaterial and fluorescent-based systems (Ye et al., 2019), many steps forward have been made in this field, and many others will be needed in the following years.

## Microrna Variants in Cancer

IsomiRs are functional and independent molecules able to bind Ago proteins and play the role of gene-expression regulators as their canonical counterparts (Cloonan et al., 2011; Londin et al., 2015; Haseeb et al., 2017). Besides, their expression is finely regulated in different tissues and pathological conditions (Linsen et al., 2009; Wyman et al., 2011).

Telonis et al. (2017) performed a comprehensive study of isomiR expression, analyzing miRNA-Seq data from 32 different normal and tumor tissues belonging to The Cancer Genome Atlas (TCGA). The authors showed that differentially expressed isomiRs could discriminate between normal and cancer tissues and different tumor types (Telonis et al., 2017). Using binarized isomiR profiles that individually classify each isomiR as “present” or “absent,” they were able to recognize and cluster several tumor datasets efficiently (Telonis et al., 2017). This study has also highlighted that some isomiRs are ubiquitously expressed while others are tissue specific, such as microRNA variants of miR-9 and miR-219, two microRNAs detected mainly in the nervous system and involved in neuronal development and differentiation, expressed only in low-grade glioma (LGG) datasets, suggesting the potential role for these cancer-specific molecules as biomarkers of some types of tumor (Telonis et al., 2017).

Other groups carried out isomiR profiling using TCGA miRNA-seq data, focusing mainly on edited microRNA expression.

Wang et al. (2017) reported the presence of 19 editing sites frequently expressed in cancer tissues, called “miRNA-editing-hotspots,” presenting an editing level above 5% and detected in at least 10 samples per cancer tissue. The analysis, conducted on 8,595 TCGA samples from 20 different tumor types, also pointed out an association between these editing hotspots and the expression of some critical oncogenes and tumor suppressors. For instance, the edited variant of miR-200b correlates with TP53 in head and neck, endometrial, and breast cancer, with NRAS and BRAF in thyroid cancer, and with CDH1 in gastric cancer (Wang et al., 2017).

Pinto et al. (2018) analyzed 10,593 miRNA-seq samples from the TCGA dataset representing 32 cancer and normal tissues. Applying stringent filters to avoid selecting molecules with an inconsiderable level of editing, they found 129 new editing sites on mature microRNA molecules, but only 55 showing an average editing level above 1% and three below but very close to 1%, in at least one out of the 32 TCGA examined tissues (Pinto et al., 2018). The expression analysis of these well-represented edited microRNAs displayed a significant lowering of the editing level in 19 of the 22 cancer tissues compared with the corresponding normal controls. This general condition of hypo-miRNA-editing in cancer suggests that miRNA-editing dysregulation could have a role in cancer progression. To confirm these results, they observed 56 patient cases, holding 26 different editing sites in 15 diverse tumor samples, and classifying patients into two groups according to their miRNA-editing levels. In line with the previous observations, better prognosis and, consequently, better patient survival were observed to be associated with higher miRNA-editing levels (Pinto et al., 2018).

In conclusion, an increasing number of papers are revealing the role of isomiRs in cancer. Conversely, the study of individual isomiRs and their specific targets is still an emerging field but of immense importance because the potential of isomiRs as prognostic and diagnostic markers in tumor conditions might be invaluable.

## Isomirs in Neurodegenerative and Metabolic Diseases

Although most of the attention has been paid to the role of isomiRs in cancer, the interest in the behavior of these newly discovered molecules in chronic conditions, such as neurodegenerative and metabolic diseases, begins to catch on.

In 2016, a study involving early- and late-stage Alzheimer’s patients revealed a significant change in 5′ miRNA isoform level between the two groups of patients (Wang et al., 2016). Interestingly, among the 47 miRNAs showing relevant differences in their 5′ variants level through the progression of the disease, 17 are actively involved in Alzheimer’s disease pathogenesis (Wang et al., 2016). Similarly, an important dysregulation of canonical miRNAs and isomiRs was observed in Huntington’s patients (Martí et al., 2010). A massively parallel small RNA sequencing analysis, performed on healthy subjects and patients, unveiled that ∼80–90% of miRNAs mapped in the human brain showed modification at 3′ end, with a predominance of nucleotide addition as the most common modification, and ∼35% presented nucleotide substitutions along the sequence (Martí et al., 2010). Moreover, this study demonstrated that the dysregulation of isomiRs presenting modifications at the 5′ end significantly alter the expression of critical genes belonging to Huntington’s disease canonical pathways (Martí et al., 2010). This observation, combined with a commonly observed co-expression of canonical miRNA and isomiRs in Huntington’s patients’ samples, suggests a cooperative role for the dysregulated isomiRs and the reference microRNAs in Huntington’s disease (Martí et al., 2010).

The identification and study of new miRNA isoforms are starting to take hold also in the field of metabolic diseases. A study on the miR-27 family genes in metabolism has recently described the functional importance of miR-27 isoforms in metabolic processes associated with diseases (Ma et al., 2019). The overexpression of miR-27b-3p and two 3′ isoforms in murine hepatocytes demonstrated the different impact of these molecules on the expression of some proteins with a critical role in metabolism: only the canonical miR-27b-3p strongly downregulates PEPCK, FAS, and SREBP1C, as well as the isomiR-27b-3p negatively affects the expression of G6PASE, CPT1A, and BMAL1, thus demonstrating the independent and distinct role of these miRNA variants in liver cells (Ma et al., 2019).

Baran-Gale et al. (2013) studied the miRNA profile in murine insulinoma cells and human beta cell and whole islets, finding an abundance of highly expressed 5′-shifted isomiRs. Then, they selected 10 microRNAs as potential regulatory hubs in type 2 diabetes, three of which are represented by 5′ isomiRs: miR-375 + 1, miR-375-1, and miR-183-5p + 1 (Baran-Gale et al., 2013). *In silico* analyses and experimental validations confirmed that *Mtpn* is regulated only by the canonical miR-375, while *Atp6v0c* and *Cdc42* are predominantly repressed by miR-375 + 1 and miR-375-1, thus promoting the functional importance of 5′-shifted isomiRs as molecules able to affect the expression of type 2 diabetes–associated genes independently (Baran-Gale et al., 2013). In contrast to 5′ and 3′-shifted isomiRs, the functional role of edited microRNAs in chronic neurodegenerative and metabolic diseases has not been currently assessed, despite the central role of ADAR proteins and RNA editing in these pathological contexts (Gan et al., 2006; Singh et al., 2007; Yang et al., 2012; Gaisler-Salomon et al., 2014; Aizawa et al., 2016; Khermesh et al., 2016). Certainly, taking into account the primary role of miRNA editing in cancer, it could represent an exciting field of study for future investigations also in other diseases.

## Discussion

In the past 20 years, microRNA research has become a primary branch of molecular biology. With the increasing employment of NextGen sequencing, the identification of new microRNA variants, sometimes even more expressed and active than the database-annotated counterparts, has helped to reassess some aspects of microRNA biology so far regarded as established, such as the role of microRNA 3′ end in the stability of target-binding and Ago2 microRNA-loading process.

However, notwithstanding the great leaps forward that isomiRs research has taken, there are still many questions to answer, and, among them, the most important: are isomiRs independent functional molecules? Despite the growing number of papers supporting the evidence of a specific role for these newly discovered molecules in assisting or preventing the activity of the canonical microRNAs, the current technical and bioinformatics limitations in predicting new individual targets, especially for non-seed-based nucleotide substitutions isomiRs, imply the idea that many of these new microRNA family members could be unnecessary or repetitive. Nevertheless, it is worth pointing out that the biogenesis of isomiRs is not a casual event but occurs under precise control. Most of these novel variants are well conserved across species and specifically expressed in some tissues and physiological or pathological conditions, including cancer, thus strengthening the hypothesis of an autonomous function.

Moreover, in the past, many researchers had to face the dilemma of a multiple, and commonly opponent, role of the same microRNA in different tissues or conditions (Banzhaf-Strathmann and Edbauer, 2014; Costa-Pinheiro et al., 2015; Wen et al., 2018; Shen et al., 2019; Rezaei et al., 2020; Xiang et al., 2020). It might be interesting to speculate if the presence of differentially expressed isoforms, aggregated and analyzed as a single microRNA, could explain the microRNA duality frequently observed.

Certainly, recognizing these microRNA variants does not question all the previous relevant discoveries about microRNA biology and genetics. Instead, it means that there are still questions to address and regulatory mechanisms to explore in the complex world of these critical regulators.

## Author Contributions

LT, RD, and GN conceived the structure of the article. LT wrote the article. RD, GN, and CMC read, edited, and approved the article and helped the discussion and correction of English writing. All authors contributed to the article and approved the submitted version.

## Conflict of Interest

The authors declare that the research was conducted in the absence of any commercial or financial relationships that could be construed as a potential conflict of interest.
